# A Longitudinal Study of Preterm Infants at 12 and 30 Months: Links Among Object Interactions, Joint Engagement, and Cognitive Development

**DOI:** 10.1111/infa.70016

**Published:** 2025-03-30

**Authors:** Qin Liu, Michelle de Haan, Kathy Chant, Kayleigh Lauren Day, Mérari Jizar Lavander‐Ferreira, Neil Marlow, Catalina Suarez‐Rivera

**Affiliations:** ^1^ University College London Great Ormond Street Institute of Child Health London UK; ^2^ School of Psychology Cardiff University Cardiff UK; ^3^ Institute for Women's Health University College London London UK; ^4^ Department of Applied Psychology New York University New York New York USA

**Keywords:** cognitive development, joint engagement, longitudinal associations, multimodal engagement, object interaction

## Abstract

Development takes place when change in one domain cascades into change in another domain. Preterm infants exhibit disruptions to their object play and the maintenance of a joint focus of attention with another person. Likewise, they tend to experience cognitive delays throughout childhood. By the developmental cascades model, early features of object play and joint engagement in preterm infants predict cognitive development. We examined longitudinal associations between real‐time individual differences in parent‐infant interactions and long‐term outcomes to explore potential developmental processes. Features of infant‐object interactions and joint engagement were coded in 20 12‐month‐old preterm infants (≤ 29 weeks of gestation) during parent‐infant free play. Infants were tested again at 30 months using the Bayley Scales of Infant and Toddler Development, Third Edition. Preterm infants spent most of their time interacting with objects at 12 months, and their parents frequently engaged in their object interactions. The frequency of infant‐object interaction bouts per minute at 12 months was negatively associated with 30‐month cognitive scores. Furthermore, the percentage of infant‐object interaction bouts in which parents practised multimodal engagement was marginally associated with 30‐month cognitive scores. We discuss the associations of infant‐object interactions and joint engagement with preterm infants' cognitive development.

## 1 Introduction

Preterm infants follow developmental trajectories that lead to high risk of neurodevelopmental delays in cognitive, motor, and language domains (Bhutta et al. 2002; Chung et al. 2020; Lobo and Galloway 2013; Zuccarini et al. 2017). In particular, learning and cognitive impairments can be detected in *extremely preterm* (< 28 weeks of gestation) and *very preterm* infants (between 28 and less than 32 weeks of gestation) in the first few months after birth (Lobo and Galloway 2013) and continue throughout early childhood into adult life (Linsell et al. 2018; Eryigit Madzwamuse et al. 2015). The developmental mechanisms that set atypical trajectories of development for these preterm infants are not known as there is a complex interaction between the environment, genetic make‐up, and the consequences of preterm birth for brain development (Malachowski and Needham 2023; Lobo and Galloway 2013).

The everyday behavior of preterm infants may offer insights into this question and is the vehicle through which their genetic and neurodevelopmental make‐up is expressed. Drawing from the developmental cascades framework, behavior creates real‐time cascades between the infant and the world that ultimately lead to the development of cognitive abilities (Malachowski and Needham 2023; Masten & Cicchetti, 2010). There has been burgeoning research indicating that manual object interaction in both typical and atypical infants is closely linked to cognitive development (Malachowski and Needham 2023). However, studies on object manual exploration in preterm infants and associations with developing cognitive abilities, remain understudied. In this paper, we examine the experiences that may occur during routine parent‐infant play and that may affect the developmental trajectory in very and extremely preterm infants. We quantify features of infant‐object interactions with their parents to measure behaviors (i.e., object interactions and joint engagement) that may be theoretically related to developmental outcomes. In this setting, research on very and extremely preterm infants serves a dual purpose: it provides important insights into the longitudinal pathways linking early experiences at 12 months to later developmental outcomes in life and helps understand the experiences in preterm infants that are predictive of future outcomes in response to the growing need of understanding developmental trajectories in this population. The estimated number of preterm births (< 37 weeks of gestation) is 47,487, which accounts for approximately 7.6% of 624,828 live births in England and Wales in 2021 (Office for National Statistics 2023), making it very important to understand development of these babies.

### 1.1 Infant‐Object Interactions in Preterm Infants

Prematurity is associated with a range of neurodevelopmental problems (Aarnoudse‐Moens et al. 2009; Woythaler et al. 2011), including delays in cognitive development (Baron and Rey‐Casserly 2010; Bhutta et al. 2002; Linsell et al. 2018), motor development (De Kieviet et al. 2009; Woythaler et al. 2011) and language development (Rabie et al. 2015). Additionally, it is also associated with a series of behavioral issues such as slow processing and inattention, alongside impairments in social cognition (Downes et al. 2018; Garner et al. 1991; Kobaş et al. 2022; Olafsen et al. 2006; Lobo et al. 2015; Rose et al. 1979). In particular, preterm infants display atypical features of object exploration compared to full‐term infants (≥ 37 weeks of gestation) during the first year of life (Lobo et al. 2015; Ruff et al. 1984; Sigman 1976; Zuccarini et al. 2016). Preterm infants exhibited developmental lags in processing visual‐haptic and exclusively visual information within the first year of life (Lobo et al. 2015; Rose et al. 1979); they showed delayed engagement in active object play (i.e., mouthing and turning/rotating) at 6 months of age (Zuccarini et al. 2016), were less able to match their exploratory behavior to object properties in the first 6 months (Lobo et al. 2015) and showed delayed responses to novel objects at 8 months of age (Sigman 1976).

The term “infant‐object interactions” refers to episodes of manual contact with an object in which infants intentionally manipulate or displace an object(s) (e.g., feeling, moving or shaking an object), and is, therefore, a form of object exploration (Herzberg et al. 2022). Only a few studies have examined object play interactions in preterm infants. Studies on preterms indicate that preterm infants, including moderate to late preterm infants (32 to less than 37 weeks of gestation), very, and extremely preterm, interact with objects in the world in unique ways (e.g., spend significantly less time in object exploration) that differ from typically developing term born infants during the first year of life (Lobo et al. 2015; Rose et al. 1979; Sigman 1976; Zuccarini et al. 2016). In contrast, full‐term infants aged 11 months to 2 years spend most of their play time (∼60%) interacting with objects (Herzberg et al. 2022; Karasik et al. 2011; Swirbul et al. 2022). Their object interaction bouts are very frequent, time‐distributed, and characterized by variable durations with objects (range = 0.03 s to 22.3 min) (Herzberg et al. 2022; Swirbul et al. 2022).

### 1.2 Joint Engagement During Object Play in Preterm Infants

An infant's manual interactions with objects provide opportunities for parents share in the focus of attention. If preterm infants experience difficulties with object play, then joint engagement of the infant and caregiver around the same object may also be affected. Laboratory‐based as well as at‐home studies of free‐flowing parent‐infant interactions with full‐term infants in their first years of life found that parents engage with the object of infant play approximately 50% of the time (Suarez‐Rivera et al. 2022; Yu and Smith 2013). Furthermore, caregivers provide rich, multimodal input to infants during spontaneous play through timely gazes, manual actions or linguistic cues during the first 2 years of life (Schatz et al. 2022; Suarez‐Rivera et al. 2022; Suarez‐Rivera et al. 2019; Yu and Smith 2013). Specifically, multimodal parental input accounts for over half of joint engagement bouts at home (Schatz et al. 2022; Suarez‐Rivera et al. 2022). According to Vygotsky's sociocultural theory (Vygotsky and Cole 1978), the infant's interactions with their more knowledgeable parents provide a significant pathway for learning and development. Thus, parental joint engagement behaviors yield valuable opportunities to scaffold infant‐object interactions (e.g., the duration of infant play bouts and the successful completion of object play tasks), infant language learning, and social development (Malachowski and Needham 2023; Schatz et al. 2022; Serra et al. 2020; Suarez‐Rivera et al. 2019; Yu et al. 2019).

No studies to date have specifically examined infant‐parent joint engagement during spontaneous, free‐flowing play among preterm infants. In this group, most of the evidence concerning joint attention abilities comes from studies outside the context of free‐flowing play. These studies demonstrate their impaired joint attention abilities (Olafsen et al. 2006), attentional control (Downes et al. 2018), and ability to adjust their attention (De Jong et al. 2015), alongside difficulties interacting with their caregivers (Garner et al. 1991). Importantly, parents of preterm infants may interact in similar ways during free‐flowing play compared with parents of term infants. Specifically, parents of preterm infants are equally likely to respond to infant behavior and thereby exhibit patterns of caregiver responsivity that do not differ from patterns of parents of full‐term infants in the first years of life (Bilgin and Wolke 2015). Thus, the understanding of joint parent‐preterm infant engagement in object interactions during spontaneous, free‐flowing toy‐play contexts remains limited.

### 1.3 Associations Between Object Play, Joint Engagement, and Cognitive Development

Features of early object interactions and the degree to which caregivers jointly engage with those objects may have profound effects on cognitive development. Object play provides infants with numerous opportunities to learn about the world in their early years (Piaget 1954). Thus, object interaction is critical for cognitive development. By manually engaging with objects, infants learn to recognize physical properties (Baumgartner and Oakes 2013); understand how to adapt and refine their actions (Gibson 2000; Soska et al. 2010); and exercise their problem‐solving faculties (Caruso 1993). A growing body of research suggests that infants learn about the object of play when they focus their attention on the object and persist in the face of distractions (Baumgartner and Oakes 2013; Oakes and Baumgartner 2012; Yu et al. 2019). Likewise, joint parent‐infant engagement in object interactions is widely recognized as crucial for developing cognitive abilities through scaffolding (Vygotsky and Cole 1978) such as maintaining or extending the duration of infant attention to a given object (Deák et al. 2018; Schroer and Yu 2022; Suarez‐Rivera et al. 2019; Yu and Smith 2016), reaching more sophisticated levels of manual engagement with objects such as pretend play (Bigelow et al. 2004; Schatz et al. 2022), and facilitating early language development (Brooks and Meltzoff 2005; Yu et al. 2019).

Despite the importance of object interactions and joint engagement for infant learning and development, only a handful of studies have examined links between object interactions, joint engagement and later cognitive abilities among preterm infants. Ruff et al. (1984) conducted structured laboratory tasks and found that the ability to manipulate objects at 9 months corrected age in preterm infants (between 26 and 34 weeks of gestation) was related to their cognitive ability at 24 months of age. Specifically, infant‐object play was measured by presenting one object for 30 s in each trial (for a total of 12 continuous trials). Preterm infants who spent more time manipulating objects at 9 months tended to have higher cognitive functions at 24 months. Building upon Ruff et al. (1984), Zuccarini et al. (2017) found a longitudinal association between specific object interactions (i.e., the duration of oral and manual exploration) by extremely preterm infants during mother‐infant free play in the laboratory at 6 months corrected age and their cognitive and language development at 24 months corrected age. Specifically, Zuccarini et al. (2017) demonstrated that extremely preterm infants who spent more time on oral and manual explorations at 6 months tended to have higher language and cognitive abilities at 24 months. These studies suggested that infant‐object play at 6 months is an important predictor of later cognitive ability and provided detailed insights into object exploration in preterm infants. However, neither of them provided a detailed characterization of infant‐object interaction bouts (e.g., the duration of each object bout per minute and the percentage of object play bouts involving a single toy), or examined the features of joint parent‐infant engagement in object interactions in the context of free‐flowing play interactions at 12 months. The lack of such studies limits the understanding of the role of infant‐object play and joint engagement in cognitive development in preterm infants.

### 1.4 Current Study

Two broader aims were pursued in this study. First, we described the social interactions around objects between preterm infants at 12 months corrected age and their caregivers. We examined infants' object interactions, focusing on properties that have been studied in term infants but not in preterms. Likewise, we quantified the joint engagement of their parents during their spontaneous play focusing on the different behaviors that parents use to engage with objects of infant action. Our description of features of object play and joint engagement during joint toy play with the caregiver in preterm infants adds to our understanding of how development happens in this population. Such everyday behaviors cascade in real time into other behaviors and over time, affect developmental trajectories. Finding behaviors of preterm infants that differ from those normally seen in the population of typically developing infants will likely be important because such behaviors may become possible targets for intervention (e.g., object interactions). In turn, behaviors that occur just as they do in typically developing infants (e.g., joint engagement with the caregiver) could be leveraged to reap the benefits of those behaviors in preterm infants. Therefore, research is needed to quantify how these behaviors play out in real time for preterm infants.

Second, we investigated associations between features of social interactions at 12 months (i.e., infant object play and joint engagement) with cognitive abilities at 30 months corrected age. If object play and joint engagement play a key role in development, then early individual differences in object play and joint engagement in preterm infants should correlate with later cognitive ability. This study's aim was to explore the longitudinal associations between infant‐object interactions, joint engagement, and cognitive development in extremely and very preterm infants.

Four research questions (RQs) guided the study:

RQ1: How frequently and for how long do preterm infants interact with objects during parent‐infant spontaneous play at 12 months corrected age, and how many objects are involved in each infant‐object play bout?

RQ2: How frequently do parents exhibit joint engagement behaviors (i.e., manual engagement, object‐related language and multimodal engagement) during infants‐object interactions at 12 months?

RQ3: Are there longitudinal associations between infant‐object interactions at 12 months and their cognitive abilities at 30 months corrected age?

RQ4: Are there longitudinal associations between parental joint engagement during infant‐object interactions at 12 months and cognitive abilities at 30 months corrected age?

Based on previous studies (Herzberg et al. 2022; Lobo et al. 2015; Swirbul et al. 2022; Zuccarini et al. 2016), we hypothesized that 12‐month preterm infants would spend less than 60% of their time interacting with objects but that the total time spent in object interaction bouts would be brief (RQ1). For RQ2, previous research indicated that mothers of preterm infants are not less sensitive or less responsive to their children than mothers of full‐term infants (Bilgin and Wolke 2015). Therefore we hypothesized that parents would frequently engage (manually, through object‐related language, or multimodally) with the object(s) of their infant's manual actions. A joint engagement rate of 40%–60%, whether generated through manual engagement, object‐related language, or a combination of the two, is considered frequent, as continuous (100%) caregiver involvement in infant‐object play is neither realistic nor essential for infants' learning and development (Suarez‐Rivera et al. 2022). For RQ3, based on previous research (Ruff et al. 1984; Zuccarini et al. 2017), we predicted significant associations between infant‐object interactions at 12 months and their cognitive abilities at 30 months. We had a non‐directional hypothesis for RQ3 given the scarcity of research describing manual object interactions during free play in this population. Therefore, we did not hypothesize which properties of bouts should be negatively or positively related to cognitive abilities. For RQ4, building on the pivotal role of caregivers' scaffolding behaviors in facilitating exploration‐related learning (Malachowski and Needham 2023; Schatz et al. 2022; Suarez‐Rivera et al. 2022), we hypothesized that parental joint engagement during infant‐object play at 12 months would be positively associated with cognitive abilities at 30 months.

## 2 Method

### 2.1 Participants

The data for this study were obtained from the University College Hospital preterm development project (UCH PDP), a large prospective cohort study on preterm infants. A total of 20 preterm infants (15 males, 75%) were included in this study; they were born at less than or equal to 29 weeks of gestation (*M* = 25.65 weeks, *SD* = 1.79 weeks, range = 23–29 weeks), and had a median birth weight of 750 g (*SD* = 153 g; range = 585–1095 g). The cohort comprised 17 extremely preterm infants (85%) and 3 very preterm infants (15%). Recruitment for this study was conducted in the Neonatal Unit at University College Hospital in London, United Kingdom. Exclusion criteria for preterm infants included a low likelihood of survival or the presence of severe congenital abnormalities. Among the neonatal complications characteristic of the included preterm infants, 5 out of 20 (25%) exhibited intraventricular hemorrhage (IVH) or periventricular injury (PVI) at grades I/II, and 5 out of 20 (25%) had IVH or PVI > grade II. The incidence of retinopathy of prematurity (ROP) at grades I/II was 9 out of 20 (45%), while 3 out of 20 (15%) had ROP > grade II. Of the 20 preterm infants, 60% (*N* = 12) were White, 20% Asian, 10% mixed African‐white, 5% African, and 5% were undisclosed ethnicity.

Free play interactions of 21 preterm infants and their parents were videotaped at 12 months corrected age. Corrected age (i.e., calculated as the number of months/days between the testing date and the infant's due date rather than their birth date) is commonly used by researchers when examining preterm infants (Johnson and Marlow 2006) to capture the level of post‐term neuropsychological maturation of preterm infants in the first years of life. One preterm infant was excluded because they rarely appeared on camera during parent‐child play at 12 months and it was not possible to obtain sufficient data from the interaction. Parents were 17 females (85%). Most parents (80%; *N* = 16) had qualifications greater than the General Certificate of Secondary Education (GSCE). The socio‐economic status of participating families was determined via their postcode's Index of Multiple Deprivation (IMD) quintile score (NPEU 2013), where 5 = most deprived and 1 = least deprived. The mean IMD quintile score among the preterm infants' families was 3.1 (*SD* = 1.3). Ethical approval was obtained from the Northwest London Research Ethics Committee. Written informed consent was obtained from all parents of preterm infants for the use of their data and their participation in the project.

The sample size provided sufficient power for the main analysis of the study. Post‐hoc power analyses of the correlation point biserial model with a sample size of *N* = 20, a two‐tailed test, and a significance level of *α* = 0.05, conducted using G*Power 3.1 (Faul et al. 2007), showed that the study had a power of 0.99 to detect the anticipated effect size of 0.7; analyses also suggested that power was 0.68 to detect the anticipated effect sizes assuming an effect size of 0.5. Furthermore, post‐hoc power analyses of the linear multiple regression with a sample size of *N* = 20, an effect size estimate of Cohen's *f* ^2^ = 0.5, a significance level of *α* = 0.05, one tested predictor, and a total of 4 predictors, showed a power of 0.84 to detect the anticipated effect size. In sum, this study was well powered with the assumption of strong effect sizes (i.e., 0.5 and 0.7). Additionally, the sample size used here is similar to the sample size used in prior research on preterm infants (Ruff et al. 1984; Zuccarini et al. 2017).

### 2.2 Procedure

**FIGURE 1:**
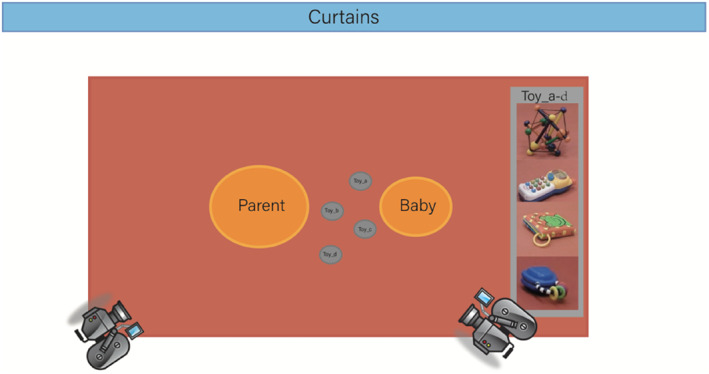
The parent and infant were seated on the mat. Four toys (i.e., one big white phone, one small blue phone, one book, and one spiky ball) were placed in front of the parent and infant. The cameras were placed on tripods.

Parent‐infant interactions at 12 months were videotaped in a quiet, infant‐friendly, and semi‐naturalistic laboratory environment. The parent and the infant were asked to sit in the middle of a red mat on which four age‐appropriate toys were placed, including one book, two toy phones, and one spiky ball (Figure 1). The parent was instructed to play with their infant using the toys as they would at home. Two cameras were placed in the room using angles that best fit the arrangement of the dyads and kept the infant's and the parent's faces visible (in profile) in the frame. Once the parent and the infant were settled and happy, the experimenter turned on the cameras and started the recording. Then, the experimenter left the parent and the infant alone. Most of the videos (85%) were around 6 min long, with recording durations ranging from 5.23 to 7.49 min (*M* = 6.5 min, *SD* = 0.52 min; 95% CI [6.26, 6.74]).

Data from both parents and infants during spontaneous, free‐flowing play at 12 months were coded using Datavyu (https://datavyu.org), a computerized coding tool commonly used to analyze user‐defined behavior (e.g., object interactions and joint engagement) frame‐by‐frame and time‐lock codes to the frames of a video.

### 2.3 Object Interactions

The start (i.e., onset) and end (i.e., offset) of each object interaction bout were marked for each infant. Onset occurred when the infant's hand(s) began to manually displace an object in space (or a combination of objects). Onsets were coded, whether an infant's hand had not yet touched an object or had already been touching one or multiple objects. That is, if the infant was already touching an object, a new onset was coded when the infant simultaneously touched a new toy. For instance, when an infant had already touched the book for a while and then proceeded to touch a telephone toy while simultaneously maintaining interaction with the book toy. The bout end was marked if the infant's hand moved away from an object for more than 3 s (Herzberg et al. 2022; Schatz et al. 2022; Suarez‐Rivera et al. 2022). For each object interaction bout, the number, and type of objects that the infant played with were coded.

### 2.4 Parent‐Infant Joint Engagement

Parental joint engagement was defined as object interaction bouts in which parents used manual touch and/or object‐related language to jointly engage with the same object that their infant was touching (Schatz et al. 2022; Suarez‐Rivera et al. 2022). Specifically, parental manual engagement was defined as the first time the parent's hand came into contact with the same object the infant was touching (Schatz et al. 2022; Suarez‐Rivera et al. 2022; Suarez‐Rivera et al. 2019). The coder watched each object interaction bout for the presence or absence of manual engagement. When an infant was touching multiple objects, the parent only needed to touch one of the objects for the action to be coded as manual engagement. Manual engagement bouts were classified as parent‐led or infant‐led. Bouts were coded as parent‐led when the parent touched the object of joint engagement first and then followed by the child who touched the object after the parent did, and as infant‐led when the infant initiated the object touch followed by the parent.

Parents were deemed to be using object‐related language when they spoke for the first time during an infant's object interaction bout about the object (or set of objects or the activity) of infant action (Suarez‐Rivera et al. 2022; Suarez‐Rivera et al. 2019). Notably, negative instructions or redirections such as “Do not touch that book” were not coded as object‐related language.

As such, infants‐object interaction bouts could be described depending on whether the parent engaged in a coded behavior as (1) having manual engagement (yes/no), (2) having object‐related language engagement (yes/no), or (3) as having multimodal joint engagement (i.e., manual and object‐related language engagement, yes/no).

### 2.5 Cognitive Abilities

The Cognitive scale of the Bayley Scales of Infant and Toddler Development (third edition) (Bayley‐III) (Bayley 2006) was used in this study to examine cognitive abilities of preterm infants at 12 and 30 months corrected age. The Bayley‐III Cognitive scale was designed to assess the cognitive, language, and motor domains of children from 1 to 42 months of age. It provides standardized cognitive composite scores, normed to a mean of 100 (*SD* = 15). A Bayley‐III Cognitive composite score below 85 points was interpreted as indicative of below‐average cognitive development. This assessment was conducted at the Clinical Research Facility, including at University College Hospital, with session durations ranging from 30 to 90 min based on the infant's age and developmental level. In our current cohort of preterm infants, the mean 12‐month Cognitive composite score was 99.69 (*SD* = 9.74, range = 85–120) and the mean 30‐month Cognitive composite score was 102.3 (*SD* = 14.1, range = 85–140). A higher Cognitive composite score on the Bayley‐III Cognitive scale indicates that the infant has a higher level of cognitive ability.

### 2.6 Inter‐Observer Reliability

Two independent coders were involved in coding the parent‐infant interaction videos in this study. The first coder annotated all videos whereas the second coder randomly selected 25% of each video to ensure inter‐observer reliability. Cohen's Kappa was calculated at the frame level of each video. The mean of Cohen's Kappa values for the presence of infant‐object interaction bouts and type of objects was 0.84 (*SD* = 0.15, range = 0.4‐1.0) and 0.92 (*SD* = 0.09, range = 0.7‐1.0), respectively. Cohen's Kappa scores for parental joint manual engagement and object‐related language codes were calculated by aggregating all data from four participants selected at random. Kappa scores ranged between 0.684 and 1.00 (*M* = 0.81, *SD* = 0.17).

### 2.7 Statistical Analysis Plan

Appropriate descriptive statistics were used to quantify features of object interactions and parental joint engagement (RQs 1 and 2). Features of object play were: frequency of object bouts per minute, proportion of time spent with objects, median duration of object bouts, proportion of bouts with one object, and total number of unique objects that the child touched. Features of parental joint engagement were: the frequency of object bouts in which parents exhibited manual engagement, object‐related language, and multimodal engagement per minute and the proportion of object bouts in which parents practised these three types of engagement, respectively.

For RQ3 and RQ4, Pearson's correlation coefficients were first computed to quantify bivariate associations between cognitive composite scores and (a) variables from 12‐month infant‐object interactions and (b) variables from parental joint engagement during 12‐month infant‐object interaction bouts. Specifically, coefficients were calculated for 12‐month‐old variables that had substantial variability within their respective distributions and for variables that were unique (i.e., not correlated with other measures). Specifically, we focused on *frequency of object play bouts per minute*, *proportion of time with objects*, and *proportion of bouts with one object at a time* as object play variables. Variables *proportion of object interaction bouts in which parents practised manual engagement*, *object‐related language*, and *multimodal engagement,* as well as *proportion of object bouts that were parent‐initiated* were used for joint engagement variables. Then, multiple linear regression analyses were performed to quantify associations between these variables and cognitive composite scores at 30 months after adjusting for infant sex, family socioeconomic status, and cognitive composite scores at 12 months. All analyses were performed using SPSS, Version 27.0, and the significance level was set at 5%.

Exploratory analyses were also conducted to examine associations of cognitive development, object play, and joint engagement simultaneously. That is, object play and joint engagement were included as predictors in the same model. Although important and parsimonious, this type of model addressed different questions and was secondary to models designed to directly test our primary hypotheses: keeping all things equal on parental joint engagement, what is the contribution of infant object play? Likewise, what is the unique contribution of parental joint engagement after adjusting for infant object play?

## 3 Results

### 3.1 Object Interactions

**FIGURE 2:**
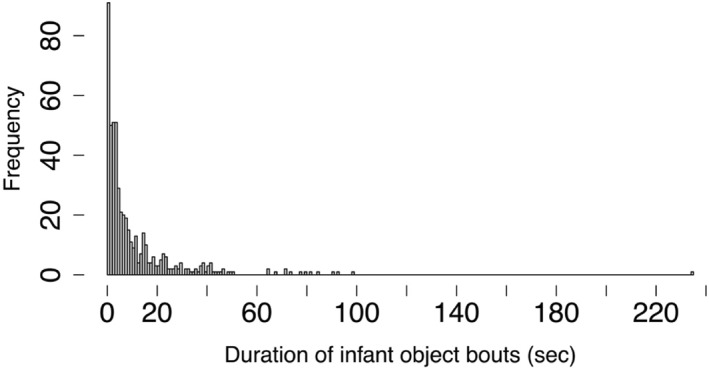
Frequency of the duration of infant‐object interaction bouts (in seconds).

Among 20 preterm infant‐parent dyads, recordings lasted 5.23–7.49 min. Infants spent most of their observation time (*Mdn* = 85.6%, *IQR* = 30.5%, range = 16.3%–97.3%) interacting with objects (Table 1). On average, they engaged in 4.15 object interaction bouts per minute (*M* = 4.15 bouts, *SD* = 1.69 bouts, range = 1.70–7.48 bouts). The duration of preterm infants' interaction bouts was brief (*Mdn* = 5.67 s, *IQR* = 6.12 s, range = 2.14–21.93 s) (Figure 2). Additionally, the average percentage of object interaction bouts in which infants interacted with 1 object was 75.3% (*SD* = 13%, range = 53%–95%).

**TABLE 1:** Characteristics of object interaction bouts at 12 Months corrected age in 20 preterm infants.

Infant‐object interaction	*M*	*SD*	*Mdn*	*IQR*	Range
Bout(s) per minute (n)	4.15	1.69	4.02	2.5	1.70–7.48
Proportion of time with objects (%)	76.1	21	85.6	30.5	16.3–97.3
Bout duration per minute (s)	7.26	5.08	5.67	6.12	2.14–21.93
Proportion of bouts with single object (%)	75.3	13	75	21.8	53–95

### 3.2 Parental Joint Engagement During Object Interaction Bouts

Parents frequently engaged with the same object(s) as their preterm infants, whether through manual engagement (*Mdn* = 35%, *IQR* = 24%, range = 17%–88%), object‐related language (*Mdn* = 56%, *IQR* = 30%, range = 15%–92%), or a combination of the two (*Mdn* = 29%, *IQR* = 15%, range = 6%–80%) (Table 2). Moreover, parents initiated joint manual engagement more frequently than their infants. On average, 68% of object interaction bouts (*M* = 68%, *SD* = 20%, range = 40%–100%) with joint manual engagement were parent‐initiated, while the remaining 32% were infant‐led. The parent was more likely to initiate joint engagement by touching objects first than to follow in on the infant‐initiated object bouts.

**TABLE 2:** Characteristics of parental joint engagement during object interaction bouts in 20 parent‐infant dyads.

Parental joint engagement	*M*	*SD*	*Mdn*	*IQR*	Range
Parental manual engagement					
Bout(s) per minute (n)	1.55	0.68	1.42	1.08	0.78–3.38
Bouts (%)	41	20	35	24	17–88
Parents‐initiated bout(s) per minute (n)	1.08	0.64	0.79	0.9	0.37–2.61
Parents‐initiated bouts (%)	68	20	67	33	40–100
Parental object‐related language					
Bout(s) per minute (n)	2.22	1.16	1.75	1.75	0.77–5.04
Bouts (%)	56	21	56	30	15–92
Parental multimodal engagement					
Bout(s) per minute (n)	1.16	0.63	1.08	0.47	0.43–3.08
Bouts (%)	32	18	29	15	6–80

### 3.3 Associations Between Object Interactions at 12 Months and Cognitive Composite Scores at 30 Months Corrected Age

**FIGURE 3:**
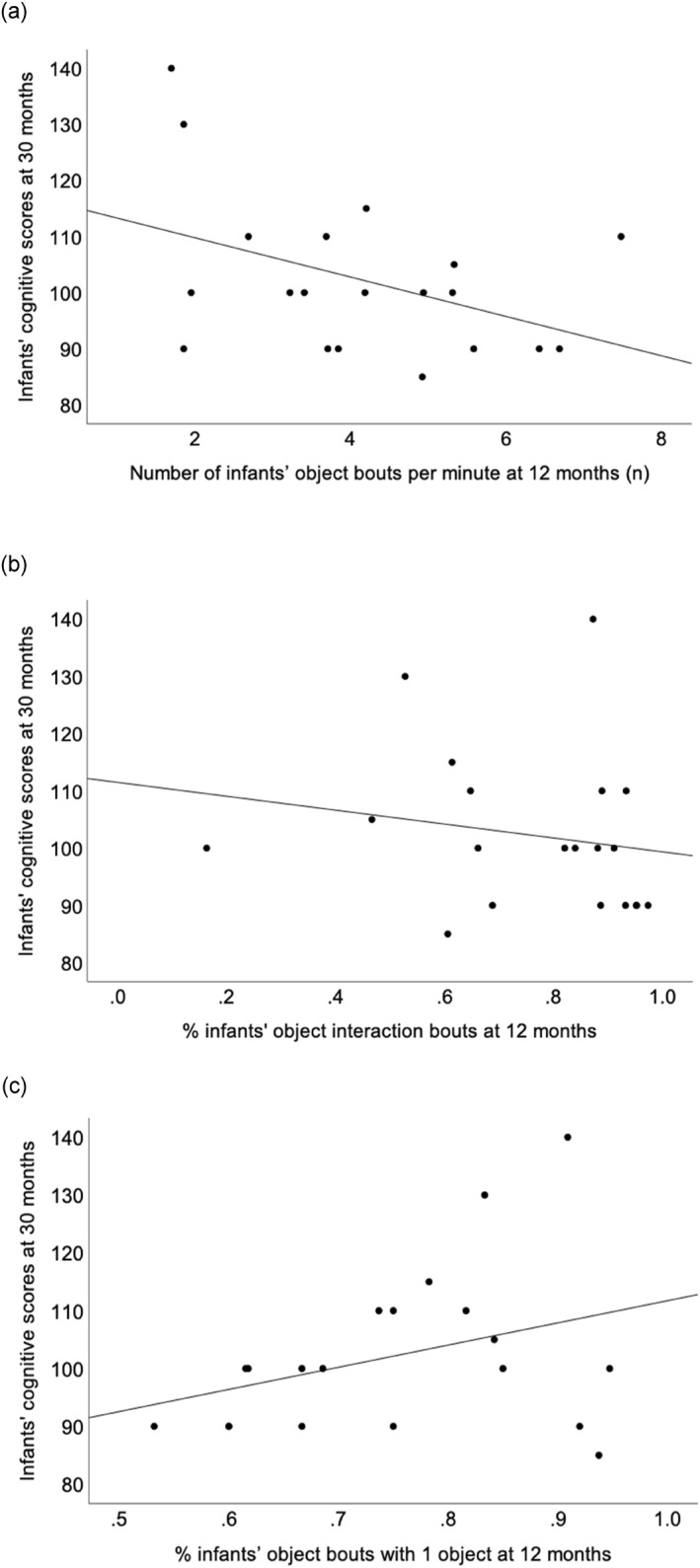
Associations between cognitive composite scores at 30 months and (a) the number of object interaction bouts per minute at 12 months, (b) the percentage of time infants spent interacting with objects, and (c) the percentage of object bouts with one object at 12 months.

Three Pearson's correlation analyses examined associations between infant‐object interactions at 12 months and cognitive composite scores at 30 months. The first indicated a moderate negative, but non‐significant association (*r* = −0.42; 95% CI [−0.73, 0.03]; *p* = 0.065) between the frequency of object interaction bouts per minute and the cognitive composite scores at 30 months (Figure 3a). Secondly, there was a weak non‐significant association (*r* = −0.18; 95% CI [−0.58, 0.28]; *p* = 0.445) (see Figure 3b) between the percentage of time infants spent interacting with objects at 12 months and their cognitive composite scores at 30 months. Finally, we found a moderate positive non‐significant association (*r* = 0.35; 95% CI [−0.12, 0.68]; *p* = 0.136) (see Figure 3c) between the percentage of object interaction bouts involving one object and the 30‐month cognitive composite scores.

To further investigate the moderate correlations observed in Figure 3a and 3c, two multiple linear regression analyses were conducted. Infant sex, family socioeconomic status, and infants' cognitive composite scores at 12 months were included in the two models to account for potential confounding effects. Results showed that at 12 months the frequency of object interaction bouts per minute was a significant predictor of their 30‐month cognitive composite scores, after adjustment, *F* (4, 11) = 2.26, *p* = 0.027, Adjusted *R* ^*2*^ = 25.2%, *B* = −6.20, 95%CI [−11.57, −0.83] (Table 3). Infants with fewer object bouts per minute tended to have greater cognitive composite scores at 30 months.

**TABLE 3:** Predicting 30‐month cognitive composite score from 12‐month object interaction.

Predictor	*b*	*SE*	*t*	95% CI	*p*
Number of infants‐object bouts per minute	−6.20	2.44	−2.54	[−11.57, −0.83]	0.027
Infant sex	15.53	9.06	1.71	[−4.40, 35.47]	0.114
IMD quintile scores	1.56	2.71	0.58	[−4.40, 7.52]	0.575
12‐month cognitive composite scores	−0.31	0.44	−0.70	[−1.27, 0.66]	0.497

In the second regression model, the percentage of object interactions involving one object was not a significant predictor of 30‐month cognitive composite scores, after adjustment, *F* (4, 11) = 1.02, *p* = 0.172, Adjusted *R* ^*2*^ = 0.6%, *B* = 47.45, 95%CI [−24.02, 118.90].

### 3.4 Associations Between Parental Joint Engagement and Cognitive Composite Score at 30 Months Corrected Age

**FIGURE 4:**
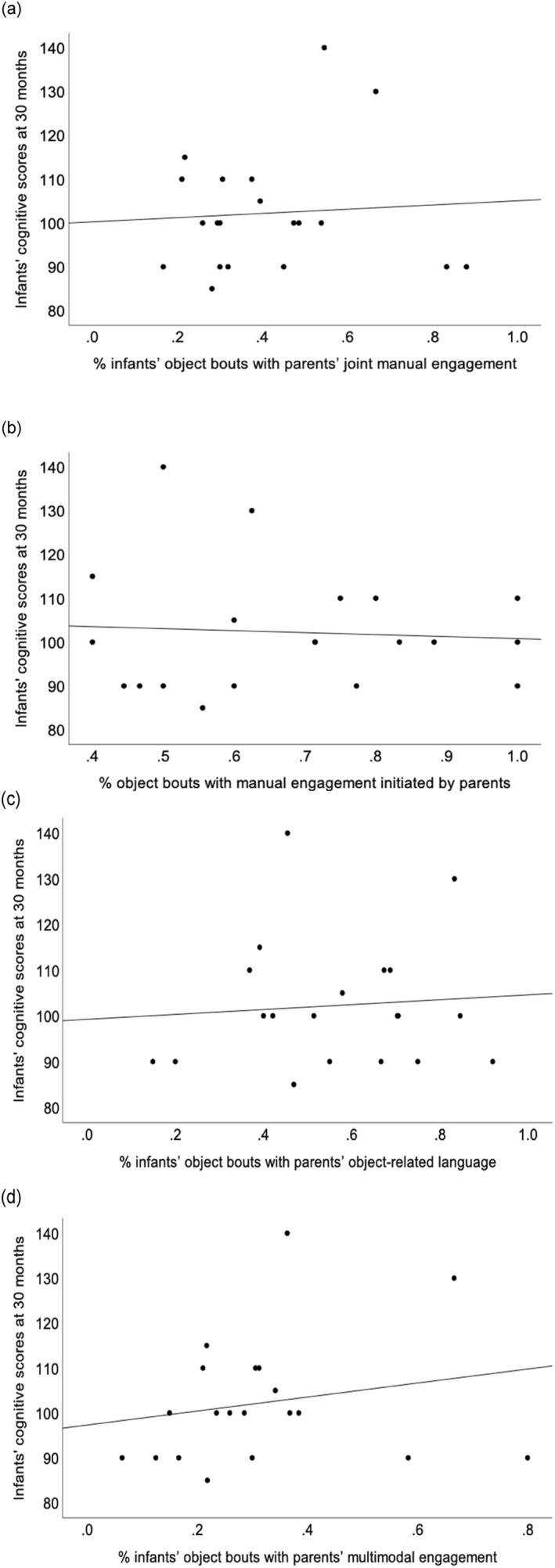
Associations between cognitive composite scores at 30 months and (a) the proportion of object bouts with parental manual engagement, (b) the proportion of object bouts with parental manual engagement initiated by parents, (c) the proportion of object bouts with parental object‐related language, and (d) the proportion of object bouts with parental multimodal engagement.

Four correlation analyses examined associations between parental joint engagement during infant‐parent object interaction bouts at 12 months and cognitive composite scores at 30 months (see Figure 4a–4d). Only one correlation analysis showed a weak, positive but non‐significant correlation between the proportion of infant‐object interaction bouts in which parents practised multimodal engagement and the cognitive composite scores at 30 months (*r* = 0.20; 95% CI [−0.27, 0.59]); *p* = 0.394) (see Figure 4d). There was no association at all for the remaining features of parental joint engagement (correlations all nearly 0 and non‐significant, all *p*'s ≥ 0.739).

Multiple linear regression analyses revealed that the percentage of infant‐object interaction bouts in which parents exhibited multimodal joint engagement at 12 months was a marginally significant and positive predictor of their cognitive composite scores at 30 months after adjustment, *F* (4, 11) = 1.66, *p* = 0.061, Adjusted *R* ^*2*^ = 0.15; *B* = 51.34, 95%CI [−2.84, 105.53] (see Table 4).

**TABLE 4:** Predicting 30‐month cognitive composite score from 12‐month parental multimodal engagement in object interactions.

Predictor	*b*	*SE*	*t*	95% CI	*p*
Proportion of infant‐object bouts with parental multimodal engagement	51.34	24.62	2.09	[−2.84, 105.53]	0.061
Infant sex	21.29	10.88	1.96	[−2.67, 45.24]	0.076
IMD quintile scores	0.72	2.80	0.26	[−5.44, 6.88]	0.802
12‐month cognitive composite scores	−0.07	0.43	−0.17	[−1.02, 0.87]	0.866

### 3.5 Exploratory Analyses

A parsimonious model examined the unique contribution of the frequency of infant object bouts per minute to cognitive development after adjusting for parental multimodal engagement. Results indicated that at 12 months, the frequency of object interaction bouts per minute was a marginally significant predictor of cognitive composite scores at 30 months after adjusting for infant sex, family socioeconomic status, cognitive composite scores at 12 months, and the proportion of infant‐object bouts with parental multimodal engagement, *F* (5, 10) = 2.81, *p* = 0.050, Adjusted *R* ^*2*^ = 37.6%, *B* = −5.15, 95%CI [−10.29, −0.01] (see Table 5). Infants with fewer object bouts per minute at 12 months had greater cognitive composite scores at 30 months after adjusting for the parental level of multimodal engagement. However, the proportion of infant object bouts with multimodal engagement was not a significant predictor of cognitive composite scores at 30 months after adjusting for infant object play (*B* = 38.88, 95%CI [−9.74, 87.49]).

**TABLE 5:** Predicting 30‐month cognitive composite score from 12‐month object interaction and parental multimodal engagement.

Predictor	*b*	*SE*	*t*	95% CI	*p*
Number of infants‐object bouts per minute	−5.15	2.31	−2.23	[−10.29, −0.01]	0.050
Infant sex	23.37	9.37	2.49	[2.49, 44.24]	0.032
IMD quintile scores	2.58	2.54	1.02	[−3.08, 8.23]	0.334
12‐month cognitive composite scores	−0.51	0.42	−1.22	[−1.44, 0.42]	0.251
Proportion of infant object bouts with parental multimodal engagement	38.88	21.82	1.78	[−9.74, 87.49]	0.105

Another parsimonious model examined the contribution of infant object play through the percentage of object interactions involving one object after adjusting for parental multimodal engagement for cognitive development. Regression coefficients for infant object play and parental multimodal engagement in this model were not significant (*F* (5, 10) = 1.41, Adjusted *R* ^*2*^ = 11.9%, *all p > 0.05).*

## 4 Discussion

The present study simultaneously quantified features of object interactions and joint engagement during spontaneous play between preterm infants and their caregivers, providing a first examination of longitudinal associations by focusing on the behaviors during parent‐infant interactions that should matter for cognitive development. This study provided two key findings. First, preterm infants spent the majority of their observation time actively engaging with objects, as evidenced by the occurrence of numerous object bouts, while their parents also frequently engaged in their infant‐object play (whether through manual engagement, object‐related language, or a combination of the two). Second, features of free‐flowing interactions during object play at 12 months in preterm infants were linked to cognitive abilities at 30 months. Specifically, infants who exhibited less frequent transitions between objects had better cognitive abilities at 30 months. Moreover, parents who engaged more frequently in multimodal behaviors (i.e., manual engagement and object‐related language) during infant‐object play marginally predicted improved cognitive outcomes in preterm infants at 30 months. We discuss the implications of findings for our understanding of object interactions in preterms, parental joint engagement, and possible cascading effects from play to cognitive development.

### 4.1 Features of Preterm Infants' Object Interactions

Our findings provide evidence for the first time that preterm infants showed brief and variable object interaction patterns during spontaneous play. That is, preterm infants spent time with objects in what seemed to be dozens of short bursts, distributed (*M* = 4.15 bouts per minute) throughout their playtime at 12 months. In addition, preterm infants engaged in play with only one object at a time in about three‐quarters of their object interaction bouts. These object interaction bouts, which focus on one object during each bout, provide preterm infants with valuable opportunities and experiences for learning, including the recognition of object‐related physical attributes, the acquisition of object control, and the practice of diverse manual actions (Baumgartner and Oakes 2013; Gibson 2000; Muentener et al. 2018).

Contrary to expectations, our study found that preterm infants actively engaged in spontaneous object interaction for the majority of the observation period at 12 months (i.e.,  ≥ 60%). There are alternative explanations for this finding. First, preterm infants may follow a developmental trajectory in which object bouts appear to be different from typical levels of play before 12 months but features of object bouts reflect those of term infants after the 12‐month mark. Indeed, our expectation that preterm infants would show low levels of object play was based on prior studies of younger preterm infants (i.e., 6 months) (Lobo et al. 2015; Zuccarini et al. 2016). Thus, it is possible the preterm infants observed here did not show decreased object interactions by 12 months because the finding that preterm infants interact less with objects occurs only for younger preterm infants, particularly during the first 6 months of corrected age (Lobo et al. 2015; Zuccarini et al. 2017). Specifically, prior findings indicated that extremely preterm infants exhibited distinct delays in motor object exploration at 6 months compared to full‐term infants; however, they appeared to recover by 9 months, demonstrating similar overall durations of motor object exploration (Zuccarini et al. 2017). This distinct developmental trajectory may be attributed to lower scores in multiple domains (e.g., psychomotor, fine motor, gross motor, and eye‐hand coordination) in extremely preterm infants at 6 months compared to full‐term infants, although these differences are no longer evident by 9 months (Zuccarini et al. 2017). As such, our sample of preterm infants may exhibit delays in object interaction during the first months of life, but these delays may not be significant by 12 months.

Another possible explanation is that the participating infants in the present study interacted a lot with objects because of the scaffolding effects that their parents had in the interaction. It is possible that parents supported infants to interact with objects through their high levels of joint engagement. Likewise, perhaps the duration of the observation period also played a role since recordings lasted between 5 and 7 min and infants could show increased levels of play during a short period. A note of caution is warranted, as this study lacked a full‐term infant comparison group, necessitating a more cautious interpretation of the characteristics of infant‐object behaviors, and future longitudinal research with preterm infants and a comparison group of full‐term infants at 6, 9 and 12 months is needed to distinguish between these possibilities and measure developmental trajectories of infant object play in preterm infants.

### 4.2 Parents Frequently Engaged in Object Interactions

A key contribution of this study is providing empirical evidence on the features of parental joint engagement during preterm infant‐parent object play. In line with the hypothesis and prior studies (Schatz et al. 2022; Suarez‐Rivera et al. 2022; Suarez‐Rivera et al. 2019; Yu and Smith 2013), our findings showed that parents frequently engaged in object interactions with their infants, with more than half of the infant‐object interaction bouts involving parent joint engagement behaviors. However, a note of caution is due here since the study did not have full‐term infants to act as a comparative group. In future investigations, it might be possible to test features of parental joint engagement in both preterm and full‐term groups in spontaneous, free‐flowing toy‐play contexts.

Furthermore, this study expands upon previous research conducted on preterm infants (e.g., Ruff et al. 1984; Zuccarini et al. 2017) by examining various features of parental joint engagement simultaneously during infant‐parent play. Specifically, the present study extended the work of Zuccarini et al. (2017), which solely focused on maternal interruption behaviors during infant‐object play, and the study by Ruff et al. (1984), which did not examine parental behaviors. Our laboratory‐based study of free‐flowing parent‐infant interactions found that parents most commonly engaged in object interaction using object‐related language, which accounted for over half of the object interaction bouts. Additionally, parents engaged in multimodal engagement, which comprised approximately one‐third of object interaction bouts, while also engaging in manual actions that accounted for approximately two‐fifths of object interaction bouts. These findings reveal the fact that parents engaged with infant‐object interactions by frequent joint engagement behaviors (whether through manual engagement, object‐related language, or a combination of the two), even when the duration of object play was only a few seconds. Importantly, the findings revealed that parents are more likely to initiate joint engagement in object play, indicating the important role of parental behaviors in infant‐object play.

There are important implications of the above findings. The interactions between infants and parents who possess greater knowledge play a significant role in facilitating infants' learning and developmental pathways (Vygotsky and Cole 1978). Specifically, parental joint engagement behaviors present significant possibilities for supporting and facilitating infant‐object interactions, infant language learning, and social development (Malachowski and Needham 2023; Schatz et al. 2022; Suarez‐Rivera et al. 2019; Yu et al. 2019).

### 4.3 Longitudinal Associations Between Object Play, Joint Engagement, and Cognitive Development

Aligned with the developmental cascades perspective, our findings show there are links between object interactions, joint engagement and cognitive abilities in preterm infants. First, the frequency of object interaction bouts was longitudinally negatively related to their 30‐month cognitive abilities, after adjustment in this group of preterm infants. Given the observed features of preterm infant‐object interactions in the study, the findings support the notion that preterm infants who had more object interaction bouts at 12 months exhibited lower cognitive abilities at 30 months. A higher frequency of infant‐object bouts per minute indicates a greater likelihood of transitioning from one object to another, resulting in less time focusing on a single object. Such findings spotlight the notion that preterm infants who exhibited “better‐organized” object play (i.e., fewer transitions per minute and increased attention on the objects they interact with) may possess enhanced cognitive abilities later in life. This aligns with the developmental cascades perspective, which emphasizes the interconnected and cumulative nature of development (Iverson 2022; Malachowski and Needham 2023). Findings indicate that object interaction behaviors during object play, which may initially seem unimportant or inconsequential, can actually have important consequences in developmental domains over time. That is, even small changes in early object interaction behaviors in preterm infants may have far‐reaching effects on their later cognitive development (Malachowski and Needham 2023).

Prior studies have suggested that specific object interaction behaviors, including infants' exuberant object play (i.e., amounts of time‐distributed and variable interactions bouts) (Herzberg et al. 2022; Swirbul et al. 2022) and the total time spent manipulating objects (Ruff et al. 1984; Zuccarini et al. 2017) may generate opportunities for later infant learning that support various developmental domains. However, these studies did not directly link all of these object interaction behaviors to infants' later developmental outcomes, particularly in preterm infants. Our findings contribute new empirical evidence supporting the effectiveness of object interactions (i.e., frequency of infant‐object interaction bouts per minute) as predictors of later cognitive abilities.

Most importantly, to our knowledge, this study has quantified features of parental joint engagement moment‐to‐moment in preterm infant‐object play for the first time and provided empirical evidence of the associations of those features with later cognitive abilities. Specifically, the study found that only *the percentage of object interaction bouts in which parents engaged in multimodal joint engagement* (i.e., manual engagement and object‐related language) was marginally associated with 30‐month cognitive abilities, after adjustment in this group of preterm infants. That is, infants who experienced frequent multimodal joint engagement with their parents, rather than only frequently experiencing manual engagement or object‐related language during object play, may exhibit better cognitive abilities at 30 months. Nonetheless, the magnitude of the association was not strong suggesting that joint engagement may not be the main vehicle cascading to learning and development (Yu et al. 2019). Instead, joint engagement may not be the relevant individual difference that predicts future development but is instead a mediator of an infant's own abilities to self‐regulate their behavior. Our exploratory findings further reveal that the marginal association diminishes when infants exhibit equal object exploration behaviors. Specifically, after controlling for individual differences in the frequency of infant object interaction bouts, *the proportion of object interaction bouts involving parental multimodal joint engagement* was no longer associated with cognitive abilities at 30 months. In contrast, the exploratory findings indicated that the frequency of object interaction bouts was still negatively related to their 30‐month cognitive abilities, even after controlling for their parental multimodal joint engagement during infant object play.

However, the correlational nature of findings limits our ability to interpret findings only through the lens of developmental cascades model. Therefore, we consider alternative explanations for these findings. For instance, it is possible that overall maturation level is predictive of both object interactions and Bayley scores. The models controlled for overall cognitive ability at 12 months, but it is not clear if this is the best way to measure overall maturation. Moreover, we did not have directional hypotheses for associations between features of infant object play and later cognitive ability. Thus, our post‐hoc interpretations may be influenced by subjective biases, highlighting the need for further studies to validate these correlations (Elliott 1996; Gelman and Loken 2013).

Likewise, even if the interpretation under developmental cascades is appropriate, the full test of the cascades model for infant object play and cognitive development requires more studies and causal inference. Future experimental studies could adopt a contrast design, wherein one group of preterm children participates in structured training or enhanced tasks designed to modify their object exploration behaviors, while another group serves as a control. Subsequent assessments of cognitive abilities in both groups would allow for testing the hypothesis that preterm children receiving object play training or enhanced tasks demonstrate significantly higher cognitive scores compared to the control group. Moreover, assessments should be conducted at multiple time points longitudinally to provide deeper insights into developmental trajectories, which are crucial for establishing causal relationships. Finally, future studies should investigate whether the longitudinal associations apply to both typical and atypical developmental pathways, thereby ensuring their generalizability across diverse populations.

### 4.4 Limitations and Future Directions

A major strength of the study is the use of a detailed frame‐by‐frame coding of infant and caregiver behavior at 12 months which provides a new description of the features of social interactions between preterm infants and their caregivers. Likewise, the study featured a longitudinal design that links timescales of development by systematically examining links among object interaction, joint engagement, and cognitive abilities of preterm infants. Nevertheless, this study has several limitations that should be considered when interpreting its findings.

First, the study sample comprised extremely and very preterm infants who did not present with severe congenital abnormalities or indicators of below‐average cognitive development. As such, findings may be generalized only to this specific range of prematurity. A further study focusing more on a broader population of preterm infants and quantifying specific levels of neurological damage is suggested. Likewise, future work may be needed to increase the sample size and increase power to detect differences. The study was sufficiently powered assuming an effect size of 0.7. Nevertheless, given assumptions of medium to low effect sizes, this study could be considered underpowered.

Generalizability may also be limited to the laboratory‐based setting. Prior studies on object interactions and joint engagement in preterm and full‐term infants have been conducted in complex contexts, including different play settings (such as spontaneous, free‐flowing play or structured task play, both at home or in the laboratory) and observation periods (e.g., 2 h or 5–15 min) (e.g., Herzberg et al. 2022; Lobo et al. 2015; Schatz et al. 2022; Suarez‐Rivera et al. 2022; Zuccarini et al. 2017). Future work is needed to validate our findings by testing these links in the home environment and longer observation periods.

Given that the study employed a coding scheme based on the manual displacement of an object frame‐by‐frame (used in prior studies Herzberg et al. 2022; Schatz et al. 2022; Suarez‐Rivera et al. 2022; Swirbul et al. 2022), the ability to generalize the findings to studies that emphasize different classifications of object exploration behaviors may be limited. Future research should develop coding schemes to capture more nuanced aspects of object exploration behavior frame‐by‐frame (e.g., precise object handling, including transferring, rotating, and fingering), as well as incorporate gross motor behaviors, which could in turn affect object exploration (e.g., posture, including sitting, lying, and standing) (Zuccarini et al. 2017).

Lastly, it should be noted that the present study did not explicitly measure the level of sustained attention and joint attention in object play despite considering the significant cascading effects of sustained attention and joint attention on later cognitive development (Lei et al., 2019; Yu et al. 2019; Suarez‐Rivera et al. 2019). Prior research with full‐term infants has used the duration of eye‐gaze (e.g., 3 s) using head‐mounted eye‐trackers to mark the presence of sustained attention (e.g., Suarez‐Rivera et al. 2019; Yu and Smith 2016; Yu et al. 2019) or changes in heart‐rate (Richards 1989). Additionally, periods during which parents and full‐term infants simultaneously fixated on the same object, measured using head‐mounted eye‐trackers, have been used to assess the duration of joint attention in object play (e.g., Yu et al. 2019; Suarez‐Rivera et al. 2019). However, eye‐trackers were not used in this study to collect eye‐gaze data. We attempted to develop alternative measures of sustained attention based on the duration of object bouts but those measures need well‐founded definitions that we did not have. For example, the threshold of 3 s developed for object looks of term infants may not be applied to the duration of bouts of object manual engagement by preterm infants. Further work is needed to develop a reliable definition of sustained attention or sustained focus for preterm infants and to explore both the role of joint attention and joint engagement in their object play. Similarly, there is abundant room for further progress in testing associations between sustained attention, joint attention, and cognitive abilities among preterm infants more broadly.

## 5 Conclusion

What do the links among object exploration, joint engagement, and cognitive development in preterm infants add to existing research? First, this study offers new empirical evidence highlighting the interconnected, cumulative, and context‐dependent nature of development. Specifically, preterm infants who engage in sustained object play and exhibit less frequent transitions between objects have later better cognitive abilities. Second, this study offers insight into the features of preterm infants' object play in the presence of caregivers. The triadic interaction, in which parents and infants manually interact with objects together, has been found to be an effective context to scaffold object interactions (Suarez‐Rivera et al. 2022). Rather than showing decreased activity, preterm infants demonstrated actively engaged in object interaction during spontaneous play, exhibiting brief and variable object interactions. Likewise, parents frequently engaged with the objects of infant play through object‐related language, manual engagement, and multimodal behaviors. Finally, this study highlights the potential causes and cascading effects of real‐time object interaction and joint engagement behaviors, which may help locate actionable targets for future interventions aimed at maximizing preterm infants' learning opportunities from object play in the first years of life. Seemingly small effects of object interaction and joint engagement among preterm infants, occurring in the first year after birth, may have cumulative effects on the development of cognitive abilities and shape developmental processes.

## Author Contributions

**Qin Liu:** conceptualization, formal analysis, methodology, writing – original draft, writing – review and editing. **Michelle de Haan:** conceptualization, data curation, project administration, resources, supervision, writing – review and editing. **Kathy Chant:** data curation, investigation, resources. **Kayleigh Lauren Day:** data curation, investigation, resources. **Mérari Jizar Lavander‐Ferreira:** data curation, investigation, resources. **Neil Marlow:** project administration, resources, writing – review and editing. **Catalina Suarez‐Rivera:** conceptualization, formal analysis, methodology, project administration, resources, software, supervision, writing – review and editing.

## Ethics Statement

Ethical approval was obtained from the Northwest London Research Ethics Committee. Written informed consent was obtained from all parents of preterm infants for the use of their data and their participation in the project.

## Conflicts of Interest

The authors declare no conflicts of interest.

## Data Availability

The data that support the findings of this study are available on request from the corresponding author. The data are not publicly available due to privacy or ethical restrictions.
